# Supplementary material for: Genomic hallmarks and therapeutic implications of G0 cell cycle arrest in cancer
Source: Genome Biol. 2023 May 23;24:128. doi: 10.1186/s13059-023-02963-4 (PMC10204193; doi:10.1186/s13059-023-02963-4)
Supplement: Supplementary file 3 — Additional file 3. Review history. [file 13059_2023_2963_MOESM3_ESM.docx]

Review History

**First round of review**

**Reviewer 1**

**Were you able to assess all statistics in the manuscript, including the appropriateness of statistical tests used?**

Yes, and I have assessed the statistics in my report.

**Comments to author:**

In this work, the authors developed a "quiescence score" based on conserved differential gene expression in previous transcriptome analysis of epithelial cells under multiple quiescence signals. They applied this quiescence score to both bulk and single-cell cancer RNA-seq datasets and showed that the score could help identify dormant or slow-cycling cancer cells. They further used machine learning to compare slow and fast cycling cancer cells and identified a set of genomic events linked with proliferation-quiescence cell fates. They also showed that under therapeutic stresses, many cancer cells switch from proliferation to quiescence. The quiescence score they developed can become a valuable tool in cancer research and potentially tracking quiescent therapeutic resistant cancer cells in the clinic, to aid the development of targeted treatment strategies in future studies.

It is important to show that the quiescence score developed in this work identifies quiescent cells ("uniquely," as the authors pointed out) but not other dormant cell types such as senescent cells. The quiescence score was indeed shown to be non-correlated with some senescent markers, such as the expression of the β galactosidase-encoding gene GLB1 and the expression of SASP genes, but a direct test would be more convincing and necessary. I wonder if the quiescence score is applied to the datasets in the paper "Unmasking Transcriptional Heterogeneity in Senescent Cells" (10.1016/j.cub.2017.07.033), whether the score can distinguish quiescent cells from senescent cells, and whether senescent cells will also show higher scores than growing cells.

Line 107, "There was a remarkably good correlation between our predicted quiescence levels and the fraction of quiescent cells." It wasn't clear how quiescence levels were predicted in the nine lung adenocarcinoma cell lines, which should be clarified in the main text. From the Methods, it appears that the quiescence scores for these cell lines were calculated using bulk RNA-seq data from the Cancer Cell Line Encyclopedia (CCLE) database. It is surely nice and interesting that the quiescence scores show good correlations with the quiescent cell fractions. Yet, A higher population-level quiescent score may indicate an overall more stable or deeper state of the quiescent (sub)population (e.g., Fig 1d), not necessarily a larger quiescent cell fraction. Can the authors provide further support at the single-cell level, e.g., by checking related scRNA-seq datasets or other means?

Similarly, it wasn't clear how the classification accuracy was determined in Fig 1b and c, which needs to be clarified in the text. In addition, for the scRNA-seq datasets in Fig 1b, when the z-score methodology is applied to individual cells, what is the correlation between the quiescence score and known cell identity (quiescence vs. proliferation) in each cell (shown in a box plot or the like)?

Minor points:

Line 48, "quiescent tumour cells can be mistaken for other therapy resistant cell types such as senescent or cancer stem cells." How do cancer stem cells become therapy resistant -- not because of quiescence? If so, they are also a subset of quiescent tumour cells, right? Please clarify.

Line 263, "We observed a good correlation between the estimates representing individual quiescence programmes and the expression of genes associated..." This statement needs to be explained in a bit more detail (e.g., how to interpret the results in Fig. 5a and b). Relatedly, in Fig 5f, how to interpret the result that the change in "generic" quiescence score before and after Palbociclib treatment seems more apparent than that in CDK4/6-inhibition "specific" quiescence score (e.g., in GSE124854)?

Line 283, "spontaneous quiescence appeared most strongly dependent on p53 functionality (Supplementary Table 4)." This statement needs to be better explained. How is p53 functionality shown in supplementary Table 4? Are the cancer types predicted to have spontaneous quiescence as the dominant quiescence form consistent with the TP53 SNV status (Fig 3c)?

Line 291, "such slow cycling, indolent tumours would have higher chances of being eradicated earlier in the disease." Why? How to reconcile this statement with those in lines 23-30 saying that quiescent cancer cells are more resistant to treatment?

Line 366, "The lack of proliferative markers such as Ki67 or CDK2 does not uniquely distinguish quiescence from other cell cycle phases, e.g., G1 or S." Why cannot Ki67 or CDK2 markers distinguish quiescence from S?

**Reviewer 2**

**Were you able to assess all statistics in the manuscript, including the appropriateness of statistical tests used?**

No, I do not feel adequately qualified to assess the statistics.

**Comments to author:**

Quiescence plays an important role in cancer progression and resistance to therapies that target actively dividing cells. In this study, the authors developed a transcriptomic signature score of quiescence that can reliably quantify quiescence state both in bulk tissue as well as in single cells. They further characterize the potential genomic constraints that may shape proliferation-quiescence decisions in solid primary tumors using regression model analysis. These findings provide a new method to uniquely evaluate cell quiescent state from transcriptomic data and identify CEP89 as a novel modulator of quiescence capacity in non-small cell lung cancer.

The results are clearly presented and visualized. And there are several major and minor comments:

1. Based on a set of genes derived from Min and Spencer (ref 26), the authors defined a Z-score method to evaluating quiescence from transcriptomic data. As shown in Figure 2c, this method outperformed classical cell cycle genes, at individual gene level, both in bulk tissue as well as in single cells. However, the expression of individual gene might not capture the real cell cycle state and several published core gene sets were commonly used to quantify the cell cycle states, such as ref 26 and Itay Tirosh et.al (Science. 2016. doi: 10.1126/science.aad0501.). It would be important to compare the present quiescent scoring method with those reported signatures.

2. The authors found that quiescent score varies significantly among different types of cancers (Figure 2b) and have different impacts on clinical outcome (Figure 6c). High cell quiescence score in patients with lung, colon or esophageal carcinoma were associated with significantly worse prognosis, while high cell quiescence score in patients with adrenal gland, kidney carcinoma or breast cancer were associated with significantly better prognosis. The apparent discrepancy should be discussed.

3. It's commonly proposed that quiescence can provide protection from cell cycle-targeting therapy and become the seed cells that mediate disease relapse. Apart from overall survival analysis as shown in Figure 6a-c, the impact of quiescence on relapse rate or cumulative incidence of relapse can be analyzed in solid and/or hematopoietic malignancies.

4. It's not justified to group patients from 31 different type of cancer using same high/low quiescent score cutoff (Figure 6a). The observed differences in prognosis may be largely due to confounding factors, such as tumor type, driver genes rather than quiescence per se.

5. The author identified TP53 integrity (not carry TP53 SNV) as one of the genomic constraints of quiescence. However, this is inconsistent with the TP53 mutations as adverse prognostic factor in cancer. The apparent discrepancy should be discussed.

6. In line 254, the suppression of CEP89 leads to an increase in quiescence in non-small cell lung cancer cell line while the higher quiescence were associated with worse survival in patients with lung cancer. The authors should discuss or provide functional evidence for the underling mechanism of how CEP89 regulating quiescence and whether CEP89 can be a potential target.

**Reviewer 3**

**Were you able to assess all statistics in the manuscript, including the appropriateness of statistical tests used?**

Yes, and I have assessed the statistics in my report.

**Comments to author:**

Driving therapy resistance by a specific cell subpopulation represents a widely accepted concept in cancer biology. However, the transcriptional differences and mutational drivers of such cell population are very poorly understood. In particular, the prevalence of resistance-associated phenotype such as quiescence is difficult to measure without reliable markers. The authors of this manuscript develop a new methodology that enables to start addressing these questions. They robustly test the use of a quiescence signature for reliable quantification of quiescent response to a variety of different compounds and in different cell types. The characterization of quiescent subpopulations will help to fill the critical knowledge gap in our understanding of the underlying mechanisms that differentiate the resistant cells from sensitive cells.

Multiple oncogenic pathways have been proposed for the therapeutic inhibition after the tumor recurrence. However, from clinical perspective, it is more important to promptly target the rare resistant cells remaining after the first-line therapy. Using bulk and single-cell RNA-sequencing data, the authors show that quiescence is associated with cell survival under targeted therapy or chemotherapy. The presented data suggest that quiescence is common in different solid tumors and can be observed as an acute response to various treatments. These are important results. The significance of this work is in identification of a robust method and signature to capture the quiescent phenotype, which can be exploited to develop strategies for inhibiting the drug resistant states. The manuscript is written clearly and concisely, the approach is robust, and the data are of high quality. As a minor comment, I only suggest to change the unconventional abbreviation PRb to phospho-RB.

In conclusion, this study nominates a transcriptional program involved in tolerance, which can be further investigated for therapeutic targeting and ultimately inhibiting therapy resistance. I give the highest recommendation for publication of this work.

**Authors Response**

**Point-by-point responses to the reviewers’ comments:**

Reviewer #1:

In this work, the authors developed a "quiescence score" based on conserved differential gene expression in previous transcriptome analysis of epithelial cells under multiple quiescence signals. They applied this quiescence score to both bulk and single-cell cancer RNA-seq datasets and showed that the score could help identify dormant or slow-cycling cancer cells. They further used machine learning to compare slow and fast cycling cancer cells and identified a set of genomic events linked with proliferation-quiescence cell fates. They also showed that under therapeutic stresses, many cancer cells switch from proliferation to quiescence. The quiescence score they developed can become a valuable tool in cancer research and potentially tracking quiescent therapeutic resistant cancer cells in the clinic, to aid the development of targeted treatment strategies in future studies.

It is important to show that the quiescence score developed in this work identifies quiescent cells ("uniquely," as the authors pointed out) but not other dormant cell types such as senescent cells. The quiescence score was indeed shown to be non-correlated with some senescent markers, such as the expression of the β galactosidase-encoding gene GLB1 and the expression of SASP genes, but a direct test would be more convincing and necessary. I wonder if the quiescence score is applied to the datasets in the paper "Unmasking Transcriptional Heterogeneity in Senescent Cells" (10.1016/j.cub.2017.07.033), whether the score can distinguish quiescent cells from senescent cells, and whether senescent cells will also show higher scores than growing cells.

*Reply: The Reviewer raises an important point, and we are grateful for the opportunity to reflect on this more thoroughly. We thank the reviewer for the suggestion of testing our scores in datasets of senescent cells from Hernandez-Segura et al – we have done this and we find that our quiescence score does separate senescent from proliferating cells in some datasets (see newly added Supplementary Figure 2a). This is in the end not surprising, as senescent cells are also arrested in the cell cycle and some pathways are likely to be shared between them. However, our score did not show good correlations with other markers of senescence in these single cell datasets, such as SASP or beta-galactosidase (see the new Supplementary Figure 2b), suggesting that the programme does not capture all features of senescence, or that some of the non-dividing cells that were isolated may not have been in a senescent but rather a deep quiescent state, which we would expect to accurately capture.*

*This exercise has helped us realise the need to more clearly phrase how our quiescence measurement is different from senescence or stemness. We are grateful for the opportunity to clarify and further demonstrate that our score captures a state of cell cycle arrest that is likely shared with other programmes such as senescence, stemness and dormancy, but is not sufficient to fully define these other states. We have updated the text in the Results and Discussion section to explain this:*

*Results lines 167-189: ”Our score showed strong negative correlations with the expression of proliferation markers (Figure 2d), suggesting that it captures a cellular state that could potentially act as a baseline for all major forms of cell cycle arrest, including stemness, senescence and clinical dormancy. Indeed, we found that our signature could to a certain extent also separate senescent cells from proliferating ones in single cell data from Hernandez-Segura et al(54), which is unsurprising since these cells are also in the G0 phase (Supplementary Figure 2a). Indeed, the authors of this resource highlight that some of the pathways uncovered in these senescent cells may be shared with quiescence. However, our score did not correlate well with other markers of senescence such as the Senescence-Associated Secretory Phenotype (SASP) and β-galactosidase activity(56-58) in single cells and even less so in TCGA samples (Figure 2d, Supplementary Figures 2b-d). We also show that the underpinning programme is distinct from that of cancer stem cells, marked by signatures associated with high telomerase activity and an undifferentiated state(59, 60) (Supplementary Figure 2e-f). This would suggest that the activation of this programme is a necessary although not sufficient condition to enable such phenotypes. Furthermore, the state we capture with our signature in TCGA is distinct from senescence or stemness.”*

*Discussion lines 488-491: “This study overcame this limitation by employing genes active in different forms of quiescence whose patterns of expression are distinct from markers of a longer G1 phase, and capture cell cycle arrest as also observed in senescence or stemness, but without overlapping with these states.”*

*Discussion lines 496-499: ”… our score could potentially also be used in a single cell setting to capture longer-lived cell cycle arrest states such as ones demonstrated in senescence or dormancy, and could assist in identifying such states with the help of additional immune or secretory biomarkers.”*

*Given that the differences between senescence and quiescence are still not very well understood at a molecular level, until we gain that understanding, it is indeed difficult to state with absolute certainty that either state can be exclusively identified. We have therefore modified our abstract so as not to make any potentially confusing claims about the uniqueness of our signature, while instead emphasising the robustness of our methodology (lines 4-5): “We developed methodology to uniquely robustly identify this state from transcriptomic signals.”*

• Line 107, "There was a remarkably good correlation between our predicted quiescence levels and the fraction of quiescent cells." It wasn't clear how quiescence levels were predicted in the nine lung adenocarcinoma cell lines, which should be clarified in the main text.

*Reply: We have now modified the sentence to clarify our approach (lines 134-137): “There was a remarkably good correlation between our predicted quiescence levels based on the expression of these cell lines from the Cancer Cell Line Encyclopedia (CCLE) and the fraction of quiescent cells in the experiment as assessed by lack of EdU incorporation over a 24h period (EdU incorporation only occurs during S phase) but particularly by lack of Rb phosphorylation.”*

• From the Methods, it appears that the quiescence scores for these cell lines were calculated using bulk RNA-seq data from the Cancer Cell Line Encyclopedia (CCLE) database. It is surely nice and interesting that the quiescence scores show good correlations with the quiescent cell fractions. Yet, A higher population-level quiescent score may indicate an overall more stable or deeper state of the quiescent (sub)population (e.g., Fig 1d), not necessarily a larger quiescent cell fraction. Can the authors provide further support at the single-cell level, e.g., by checking related scRNA-seq datasets or other means?

*Reply: We have included additional data from live-imaging experiments to show the length of the G0/G1 in individual cells in two of the nine cell lines where we have labelled cells with mRuby-PCNA to follow the length of the cell cycle phases. A graph showing these results is now included as an additional Supplementary Figure 1e. During the 48h imaging period, all G0/G1 cells do enter S-phase, albeit with variable timing. It is possible that this variable timing reflects different depths of quiescence, although since the longest G0/G1 phase in either cell line was <40h, it does not appear that there is a small subset of cells in a very deep or stable quiescent state. Therefore, we think that our quiescence score reflects a larger quiescent cell fraction rather than a subset of cells in a deeper quiescent state. However, given that the strength of our score also seems to correlate with the depth of quiescence as shown from analyses of serum starved embryonic fibroblasts (Fig 1d), we cannot exclude the possibility that the score might capture some features of deep quiescence in other cancers and even in lung cancer in certain contexts. We have no knowledge of other models available where we could further test this beyond the ones already examined, but this is an important aspect which we hope to be further elucidating in the future. We have expanded the results section with the experiments described and the discussion to comment on this current limitation of the scoring method, but also to highlight the potential to make use of the score’s versatility in capturing both parameters.*

*Results section lines 123-133 describing new experiments: “In these assays, cells were pulse-labelled with the nucleotide analogue, EdU, for 24h before fixation and immunostaining. Only cells which have proliferated in the last 24h will be labelled by EdU. EdU negative cells are classed as quiescent. Cells that were negative for phospho-Rb were also defined as quiescent since they have not yet passed the restriction point (phospho-Rb negative; see Methods, Figure 1e-f). This quiescent fraction was further validated in A549 and NCI-H1944 cells where endogenous PCNA has been labelled with an mRuby fluorophore to enable tracking of cell cycle phase lengths by live-cell imaging (Zerjatke et al(53), Methods). By quantifying the G0/G1 length in individual cells (i.e. time taken to enter S-phase after mitotic exit) over a 48h period, we could see that these cells were quiescent and not senescent (or in deep quiescence), as all G0/G1 cells did eventually enter S-phase, albeit with variable timing (Supplementary Figure 1e). ”*

*Methods section lines 699-710 describing new experiments: “Endogenous PCNA was labelled at the N-terminus with a cDNA encoding mRuby in both A549 and NCI-H1944 cells, using AAV-mediated gene-targeting, according to methods described in Zerjatke et al(53). mRuby-expressing cells were sorted into 50:50 conditioned:fresh media at single-cell density into 96-well plates by FACS and single-cell clones expanded. For live-cell imaging, 500 cells in phenol-red free media were plated per well of a 384 CellCarrierUltra plate (PerkinElmer) the day before imaging. Prior to imaging, a breathable membrane was applied to the plate and cells were imaged on the Operetta HCS microscope (PerkinElmer) at 37 °C, 5% CO2 using the 20x N.A. 0.8 objective and at 10-12 min intervals for 48h. Images were then exported and G0/G1 length (time from mitotic exit to S-phase entry) was analysed manually in FIJI.”*

*Discussion highlighting limitations and potential use (lines 493-499): “Within bulk tissue, we are limited in our capacity to distinguish between large fractions of cells residing in quiescence and a smaller fraction of cells that are in deeper quiescence, as our score seems to reflect both parameters to a certain extent. Bearing in mind this limitation, our score could potentially also be used in a single cell setting to capture longer-lived cell cycle arrest states such as ones demonstrated in senescence or dormancy, and could assist in identifying such states with the help of additional immune or secretory biomarkers.”*

• Similarly, it wasn't clear how the classification accuracy was determined in Fig 1b and c, which needs to be clarified in the text.

*Reply: We have added the following sentence to clarify this in the text (lines 99-103): “We tested the performance of our signature and scoring methodology, as well as that of other commonly used gene signatures, in distinguishing between the truly quiescent and truly proliferating cells in these seven datasets while varying the expression cut-offs for labelling cells as quiescent/proliferating based on the respective signature. ”*

• In addition, for the scRNA-seq datasets in Fig 1b, when the z-score methodology is applied to individual cells, what is the correlation between the quiescence score and known cell identity (quiescence vs. proliferation) in each cell (shown in a box plot or the like)?

*Reply: We have added a figure to highlight how quiescence score distributions differ by cell identity using box plots as suggested by the Reviewer (Supplementary Figure 1c) and have updated the text accordingly to comment on the results (lines 105-107): “Indeed, the individual cells that had been identified as quiescent in the experiments showed a significantly higher Z-score than the dividing cells across all datasets (Supplementary Figure 1c). “*

Minor points:

• Line 48, "quiescent tumour cells can be mistaken for other therapy resistant cell types such as senescent or cancer stem cells." How do cancer stem cells become therapy resistant -- not because of quiescence? If so, they are also a subset of quiescent tumour cells, right? Please clarify.

*Reply: The reviewer raises an important and complex point. While our text was not clear enough in the manuscript, our intention was to convey that indeed both senescent and cancer stem cells have cell cycle arrest as a baseline programme. In the case of cancer stem cells, this is indeed reversible and thus quiescence is a feature of these cells. We have now altered the text to clarify that we do not believe quiescence and stemness to be mutually exclusive, and together with the additional analysis of senescence suggested above, we propose that our signature of quiescence could capture a baseline cell cycle programme for all these states. With regards to therapy outcomes, it is possible that quiescent cells emerge driving drug tolerance without a stemness or senescence phenotype, as it is possible that stemness or senescence are the drivers of resistance in other contexts. To what extent one situation is more frequent than other needs further investigation. We have expanded the introduction to include these points (lines 52-59): “Even though the same cell cycle arrest programme underlies all of these states, the emergence of therapeutic resistance is differently governed in each case, e.g. short-lived via quiescence versus long-lived via senescence (10, 33-35). Increasing evidence from the literature points towards the rapid adaptation of tumour cells to drug treatments being enabled by a slow dividing or quiescent state that persists for a short period of time before the cells start reproliferating(36). Thus, quiescence could more frequently be encountered at the early stages of therapeutic resistance compared to other cell cycle arrest phenotypes.”*

• Line 263, "We observed a good correlation between the estimates representing individual quiescence programmes and the expression of genes associated..." This statement needs to be explained in a bit more detail (e.g., how to interpret the results in Fig. 5a and b).

*Reply: We have further expanded the text so as to provide more detail on what is being depicted (lines 325-330): “Specifically, strong inverse correlations were seen between our CDK4/6 inhibition scores and the mean expression of CDK4 and CDK6, or between our MEK inhibition scores and the expression of genes involved in the MAPK pathway(89). Spontaneous quiescence and serum starvation scores were most correlated with the activity of p21, or of genes involved in the cellular response to starvation, respectively. The contact inhibition programme was also captured, but with lesser specificity.”*

• Relatedly, in Fig 5f, how to interpret the result that the change in "generic" quiescence score before and after Palbociclib treatment seems more apparent than that in CDK4/6-inhibition "specific" quiescence score (e.g., in GSE124854)?

*Reply: The generic quiescence score is more broadly applicable/translatable across multiple cancer types as demonstrated in our single cell validation datasets. This could be a reason for its superior performance, although it is of course also expected that it should perform well regardless of the actual quiescence form because it was designed to do so. We would like to reiterate that the CDK4/6 inhibition score still shows highly significant differences, and outperforms all other quiescence subtype scores. We have expanded the Results section to highlight these aspects (lines 333-341): “The fact that the estimates for a generic quiescence phenotype were equally or, in some cases, more discriminative of cells treated with Palbociclib confirms the generalisability of this score, which may be more broadly applicable to different tissues and/or model systems, as shown previously in the single cell validation data. The CDK4/6 inhibition scores outperformed all the other quiescence subtype scores, suggesting that a combination of individual programmes and the generic score might best identify a specific stimulus driving quiescence.”*

• Line 283, "spontaneous quiescence appeared most strongly dependent on p53 functionality (Supplementary Table 4)." This statement needs to be better explained. How is p53 functionality shown in supplementary Table 4?

*Reply: We thank the Reviewer for highlighting the lack of clarity in Supplementary Table 4. The point we were trying to make here is that some types of quiescence appear to be more dependent on TP53 functionality than others (in other words, p53 proficiency may not be strictly necessary for some forms of quiescence). However, we acknowledge the results presented in Table 4 were difficult to read and interpret in the form provided. We have now removed this table entirely and replaced it by a visual representation of the same information by cancer type in Supplementary Figure 6g. Following the Reviewer’s suggestion in the next question below, we have also added a new Supplementary Figure 6f summarising the relation between p53 proficiency and quiescence subtype pan-cancer. These figures demonstrate that a functional (WT) p53 is most frequently enriched in a spontaneous quiescence context. We have also updated the text in the Results to further explain these findings (lines 355-361): “Some of the differences observed might be explained by the dependency between p53 activity and the form of quiescence that is enacted. Amongst the five different forms, spontaneous quiescence appeared most strongly dependent on p53 functionality, with a nearly two-fold enrichment of p53 proficient tumours in this group (Supplementary Figure 6f). Indeed, significantly higher levels of spontaneous quiescence were observed in the majority of cancers (56%) when p53 was functional rather than mutated. The second most dependent state was that of CDK4/6 inhibition, with increased levels in 36% of cancer types displaying p53 proficiency (Supplementary Figure 6g). ”*

• Are the cancer types predicted to have spontaneous quiescence as the dominant quiescence form consistent with the TP53 SNV status (Fig 3c)?

*Reply: We thank the Reviewer for the suggestion to test this. We have now done this as explained in the previous question, and have added Supplementary Figure 6f to present the results. We confirm that there is an near two-fold enrichment of p53 wild type cases in the cancers where spontaneous quiescence is the dominant form (lines 356-361): “Amongst the five different forms, spontaneous quiescence appeared most strongly dependent on p53 functionality, with a nearly two-fold enrichment of p53 proficient tumours in this group (Supplementary Figure 6f).”*

• Line 291, "such slow cycling, indolent tumours would have higher chances of being eradicated earlier in the disease." Why? How to reconcile this statement with those in lines 23-30 saying that quiescent cancer cells are more resistant to treatment?

*Reply: We acknowledge this is a conundrum but have expanded the test to discuss this in more detail and propose potential explanations. Firstly, by examining the relation between quiescence and risk of relapse as suggested by Reviewer 2 (Supplementary Figure 7b-e), we strengthen our point that significant levels of quiescence within primary tumours appear favourable to patient outcome, potentially because they are slower growing and could even be eradicated through surgery before additional therapies. We also notice favourable outcomes in specific cancers like kidney and adrenocortical carcinomas, which tend to have higher levels of primary tumour quiescence. The opposite is found in some of the lesser quiescent cancers, which could indicate there exists a threshold beyond which quiescence is favourable to patient survival by slowing down tumour growth, but below which it may enable rare persister phenotypes after treatment. Furthermore, the resistance phenotype could be acquired, rather than pre-existing, which we have already acknowledged in the Discussion previously. In all of this, we are aware that quiescence is one of the many factors that contribute to patient outcome and investigating all such known and unknown factors is a complex endeavour that requires further research. We highlight these limitations in the Discussion (lines 535-562): “Our signature of quiescence is prognostic and marks primary tumours with a lower proliferative capacity before treatment, but we also clearly demonstrate that it can be employed to track resistance to multiple cell cycle, kinase signalling and epigenetic targeting regimens, where it often appears as a short-lived phenotype. While this discrepancy may appear incompatible at first glance, it is not unlike other cellular processes that have been shown to present dual roles in a cancer setting, such as reactive oxygen species(108), but also p38α(109) or NRF2(110), both of which have been implicated in quiescence or dormancy(111, 112). It is possible that there is a tipping point between quiescence acting beneficially or detrimentally during tumour development and treatment. Furthermore, this is likely influenced by a myriad of other complex factors that we have not had the chance to analyse in depth here, and in some cases it may just be the baseline for acquiring cancer cell stemness or senescent properties. While we acknowledge this conundrum requires further study, we believe this phenotype also offers a unique opportunity to further understand mechanisms of tumour resistance.”*

• Line 366, "The lack of proliferative markers such as Ki67 or CDK2 does not uniquely distinguish quiescence from other cell cycle phases, e.g., G1 or S." Why cannot Ki67 or CDK2 markers distinguish quiescence from S?

*Reply: The reviewer is right that Ki67 and CDK2 can generally distinguish G0/G1 from S, although CDK2 could drop in expression upon DNA damage during S phase too. To avoid ambiguities, we have altered the sentence in the results to remove any statement about the S phase. The value of our score is that beyond distinguishing cells arrested in the cell cycle from the S phase, it can also distinguish G0 from G1, which is a more difficult problem. We have added a few sentences to explain this in more detail in the Discussion (lines 475-481): “The lack of proliferative markers such as Ki67 or CDK2(29, 99) does not uniquely distinguish quiescence from other cell cycle phases, e.g. G1. Miller et al(30) have shown that the Ki67 is expressed at the mRNA level but the protein is degraded continuously both in G0 and G1, and it rather acts as a graded marker of S/G2/M. Similarly, CDK2 activity is low in G0, G1, builds up at the restriction point, is high in the S phase and is then replaced by CDK1 in mitosis. Reduced CDK2 expression can manifest due to quiescence, mitosis, but also to DNA damage(102), and thus cut-offs to uniquely distinguish its activity in G0 would be difficult to define.”*

Reviewer #2:

Quiescence plays an important role in cancer progression and resistance to therapies that target actively dividing cells. In this study, the authors developed a transcriptomic signature score of quiescence that can reliably quantify quiescence state both in bulk tissue as well as in single cells. They further characterize the potential genomic constraints that may shape proliferation-quiescence decisions in solid primary tumors using regression model analysis. These findings provide a new method to uniquely evaluate cell quiescent state from transcriptomic data and identify CEP89 as a novel modulator of quiescence capacity in non-small cell lung cancer.The results are clearly presented and visualized. And there are several major and minor comments:

1. Based on a set of genes derived from Min and Spencer (ref 26), the authors defined a Z-score method to evaluating quiescence from transcriptomic data. As shown in Figure 2c, this method outperformed classical cell cycle genes, at individual gene level, both in bulk tissue as well as in single cells. However, the expression of individual gene might not capture the real cell cycle state and several published core gene sets were commonly used to quantify the cell cycle states, such as ref 26 and Itay Tirosh et.al (Science. 2016. .). It would be important to compare the present quiescent scoring method with those reported signatures.

*Reply: We thank the Reviewer for highlighting these important signatures from Tirosh et al which we had originally missed to include in our validation. Our own signature is heavily based on the differentially expressed genes from ref 26, and other markers used for validation in that paper include CDK2 or DREAM targets, which we have already compared our score to. We have now updated our analysis to also include a comparison of our scores with the G1/S and G2/M signatures from Tirosh et al, and have amended Figure 1c accordingly. We see that while the two new signatures perform quite well in distinguishing between quiescent and proliferating cells, our signature still outperforms them. We have also altered the text to reflect this (lines 108-112): “It also outperformed classical cell cycle and arrest markers, such as the expression of targets of the DREAM complex, CDK2, Ki67 and of mini-chromosome replication maintenance (MCM) protein complex genes - which are involved in the initiation of eukaryotic genome replication, as well as recently defined G1/S and G2/M signatures (50) (Figure 1c).”*

2. The authors found that quiescent score varies significantly among different types of cancers (Figure 2b) and have different impacts on clinical outcome (Figure 6c). High cell quiescence score in patients with lung, colon or esophageal carcinoma were associated with significantly worse prognosis, while high cell quiescence score in patients with adrenal gland, kidney carcinoma or breast cancer were associated with significantly better prognosis. The apparent discrepancy should be discussed.

*Reply: The discrepancy highlighted by the Reviewer is indeed puzzling. A recent study from the Espinoza lab has shown that p53 wild type colorectal cancer cells residing in a quiescence state tend to acquire a fetal phenotype that is linked with metastasis and poor outcomes, in line with our observations on prognosis for this cancer. Still, this does not explain why different cancer types would show different outcomes. We can attest that this is not due to a confounding effect from the varying stages of the cancers tested, as we have corrected for tumour stage in our model. It is possible that the different prognostic outcomes could be linked with different treatment modalities applied in these cancers, but unfortunately the treatment data in TCGA is not complete enough or sufficiently annotated to allow for testing this. It is noticeable that the cancers that have better outcome when quiescence is high tend to be amongst the ones that more frequently display quiescence, while oesophageal, colon and lung cancers present quiescence less frequently than average. Thus, another explanation for the discrepant outcomes could be the intrinsic proliferative capacity of these tumours, and perhaps quiescence levels need to be high enough to delay tumour growth otherwise they become detrimental to the patient. We have further commented on this aspect in the text, and highlight the need for further research into the topic (lines 380-402): “The outcomes also vary by tissue: when the cut-offs between high quiescence and high proliferation were defined on an individual cancer basis rather than pan-cancer, we found that lung, colon or esophageal carcinoma patients displayed significantly worse prognosis in the context of high tumour cell quiescence (Figure 6c, Methods). Indeed, p53 wild type colorectal cancers expressing a quiescence-linked fetal phenotype have been recently associated with metastasis and poor prognosis(95). In contrast, adrenocortical and kidney papillary cell carcinoma ranked in the top of cancers with improved survival. It is noticeable that the cancers in the former, worse prognosis group are also amongst the ones displaying lower than average quiescence (Figure 2b), so the observed inferior outcomes could in part be linked to these cancers being intrinsically faster progressing. It is possible there is a lower limit below which quiescence stops being useful for delaying growth and becomes detrimental instead, perhaps in conjunction with treatment. Indeed, other factors such as the type of therapy received could play a role too. While we are limited in the investigation of such factors in TCGA due to the incomplete records available, these discrepancies should be subject to future research.”*

*We also further comment on this in the discussion, emphasising the apparent dual faceted nature of quiescence in terms of its impact on outcome and our current limitations in understanding it (lines 549-558): “While this discrepancy may appear incompatible at first glance, it is not unlike other cellular processes that have been shown to present dual roles in a cancer setting, such as reactive oxygen species(108), but also p38α(109) or NRF2(110), both of which have been implicated in quiescence or dormancy(111, 112). It is possible that there is a tipping point between quiescence acting beneficially or detrimentally during tumour development and treatment. Furthermore, this is likely influenced by a myriad of other complex factors that we have not had the chance to analyse in depth here, and in some cases it may just be the baseline for acquiring cancer cell stemness or senescent properties. While we acknowledge this conundrum requires further study, we believe this phenotype also offers a unique opportunity to further understand mechanisms of tumour resistance.”*

3. It's commonly proposed that quiescence can provide protection from cell cycle-targeting therapy and become the seed cells that mediate disease relapse. Apart from overall survival analysis as shown in Figure 6a-c, the impact of quiescence on relapse rate or cumulative incidence of relapse can be analyzed in solid and/or hematopoietic malignancies.

*Reply: We have investigated the association between our quiescence signature and available relapse data in solid cancers from TCGA, METABRIC (breast cancer) and CPTAC (colon adenocarcinoma), and provide this new analysis in Supplementary Figures 7b-e. We generally find that quiescence is not strongly associated with risk of relapse, and if anything, it may be protective. This may be because there is a better chance to fully eradicate slower growing cancers which present higher levels tumour cell quiescence, and relapse is less likely to occur in these cases. We have commented on this in lines 372-377: “No clear relation was observed between quiescence levels within the primary tumour and risk of relapse, although higher quiescence was occasionally deemed favourable to avoiding disease recurrence or progression (Supplementary Figure 7b-e). Indeed, such slow cycling, indolent tumours would have higher chances of being eradicated earlier in the disease, which is consistent with reported worse prognosis of patients with higher tumour cell proliferation rates and less stable, more mutated genomes(94).”*

*We may see a different picture in hematopoietic malignancies, but we have not investigated this here as our signature has not been designed to capture quiescence in these cancers and we believe better markers are required before this state can be suitably assessed in this disease context.*

4. It's not justified to group patients from 31 different type of cancer using same high/low quiescent score cutoff (Figure 6a). The observed differences in prognosis may be largely due to confounding factors, such as tumor type, driver genes rather than quiescence per se.

*Reply: The Reviewer raises two important points here: (1) is the same cut-off between high proliferation and quiescence valid for all cancers; and (2) other confounding effects might impact prognosis and they should be accounted for.*

*(1): To account for the possibility that the split might not be optimal for every cancer, we have indeed also repeated the survival analysis by cancer type, with cut-offs between proliferation and quiescence optimised for each cancer type such that they reflect the largest prognostic difference. The significant associations are shown in Figure 6C. We acknowledge that the procedure was not clear in the text and have amended the Results and Methods sections to explain this:*

*Results lines 380-384: “The outcomes also vary by tissue: when the cut-offs between high quiescence and high proliferation were defined on an individual cancer basis rather than pan-cancer, we found that lung, colon or esophageal carcinoma patients displayed significantly worse prognosis in the context of high tumour cell quiescence (Figure 6c, Methods). “*

*Methods lines 833-835: “The optimal quiescence score cut-off value of 2.95 was determined pan-cancer using the surv_cutpoint function. We also used this function to determine optimal cut-offs for individual cancer types, as presented in Figure 6c.”*

*(2): To account for potential confounders like cancer type, stage etc, we had already included a multivariate Cox proportional hazards model in the supplementary material (Supplementary Figure 7a). We have also further clarified this in the text now (lines 370-372): “Overall, tumour cell quiescence appears to be beneficial for the long-term outcome of cancer patients, even when accounting for potential confounders such as stage, sex and tissue (Figure 6a, Supplementary Figure 7a).”*

*Finally, we acknowledge that other factors beyond the ones we are able to test in this setting could play a role in lines 400-402: “Indeed, other factors such as the type of therapy received could play a role too. While we are limited in the investigation of such factors in TCGA due to the incomplete records available, these discrepancies should be subject to future research.”*

5. The author identified TP53 integrity (not carry TP53 SNV) as one of the genomic constraints of quiescence. However, this is inconsistent with the TP53 mutations as adverse prognostic factor in cancer. The apparent discrepancy should be discussed.

*Reply: Our analyses indeed indicate that quiescence occurs mostly in a genomic background where p53 is functional, and this confirms the model proposed in the literature of p53 being a master regulator of quiescence. We also see that cancers with higher levels of quiescence (occurring mostly in a p53 wild type background) are linked with better overall survival, while cancers that have p53 mutations generally tend to be faster proliferating and have worse outcomes. This is in accordance with p53 mutations being reported as an adverse prognostic factor in cancer. However, the situation gets more complicated in the context of treatment. We provide an example where p53 wild type and mutant cell lines are treated with chemotherapy (Supplementary Figure 10 a-b). While in the p53 wild type case we see that cells are able to enter quiescence and this leads to therapeutic resistance, the quiescence phenotype is not pervasive in the mutant p53 lines. However, the mutant p53 lines are also resistant, which suggests that in this case resistance is driven by a different mechanism (e.g. by facilitating the fixation of additional resistance mutations). Therefore, resistance can develop both in a p53 wild type and mutant background, but the mechanisms are different, and the former is more likely linked to quiescence. We have now clarified this in the Results section (lines 416-421): “ This implies that although TP53 mutations confer a more aggressive tumour phenotype and may drive resistance via other mechanisms, TP53 wild-type tumour cells are more likely to be capable of entering a quiescent “persistent” state associated with drug resistance. SW480 cells showed higher apoptotic activity following treatment compared to RKO cells, particularly within actively cycling cells, further corroborating that cells capable of entering quiescence may be intrinsically less vulnerable to this therapy (Supplementary Figures 8a-b).”*

6. In line 254, the suppression of CEP89 leads to an increase in quiescence in non-small cell lung cancer cell line while the higher quiescence were associated with worse survival in patients with lung cancer. The authors should discuss or provide functional evidence for the underling mechanism of how CEP89 regulating quiescence and whether CEP89 can be a potential target.

*Reply: The worse survival in patients with lung cancer was in lung squamous cell carcinoma (LUSC) and not in lung adenocarcinoma (LUAD), the latter of which we tested here. We have now also added text to the Discussion describing two possible mechanisms for how CEP89 regulates quiescence and how it would be best to target these functions (rather than CEP89 itself) – lines 518-529: “A recent paper describes how increased CEP89 copy number and expression correlates with a worse prognosis in ovarian cancer(103), which we hypothesise could be linked to Cep89’s role in modulating quiescence. Although we do not yet know how Cep89 regulates quiescence, two roles have been ascribed to Cep89 which could be significant. First, Cep89 is required for primary cilium assembly(104, 105). The primary cilium acts as a signalling hub, transducing extracellular signals to intracellular signalling networks, many of which regulate growth and proliferation(106). Cep89 deficiency also leads to defects in Complex IV assembly in the electron transport chain in mitochondria, leading to decreased mitochondrial function and ATP production(107). Decreased ATP would impair the ability of cells to proliferate. Since Cep89 is a coiled-coil protein with no obviously targetable regulatory domains, it will be important to ascertain which Cep89 function is key to regulating the balance between proliferation and quiescence in cancer cells to be able to potentially target that process, rather than Cep89 itself, to induce or maintain quiescence. ”*

Reviewer #3:

Driving therapy resistance by a specific cell subpopulation represents a widely accepted concept in cancer biology. However, the transcriptional differences and mutational drivers of such cell population are very poorly understood. In particular, the prevalence of resistance-associated phenotype such as quiescence is difficult to measure without reliable markers. The authors of this manuscript develop a new methodology that enables to start addressing these questions. They robustly test the use of a quiescence signature for reliable quantification of quiescent response to a variety of different compounds and in different cell types. The characterization of quiescent subpopulations will help to fill the critical knowledge gap in our understanding of the underlying mechanisms that differentiate the resistant cells from sensitive cells.

Multiple oncogenic pathways have been proposed for the therapeutic inhibition after the tumor recurrence. However, from clinical perspective, it is more important to promptly target the rare resistant cells remaining after the first-line therapy. Using bulk and single-cell RNA-sequencing data, the authors show that quiescence is associated with cell survival under targeted therapy or chemotherapy. The presented data suggest that quiescence is common in different solid tumors and can be observed as an acute response to various treatments. These are important results. The significance of this work is in identification of a robust method and signature to capture the quiescent phenotype, which can be exploited to develop strategies for inhibiting the drug resistant states. The manuscript is written clearly and concisely, the approach is robust, and the data are of high quality. As a minor comment, I only suggest to change the unconventional abbreviation PRb to phospho-RB.

In conclusion, this study nominates a transcriptional program involved in tolerance, which can be further investigated for therapeutic targeting and ultimately inhibiting therapy resistance. I give the highest recommendation for publication of this work.

*Reply: We thank the Reviewer for the positive comments and enthusiasm for our work. We have now altered the phospho-Rb abbreviation throughout the manuscript and in Figures 1e-g, 4f and 7b according to the Reviewer’s suggestion.*

**Second round of review**

**Reviewer 1**

The authors have addressed most of my previous technical questions. However, one critical issue remains. In the revision, the authors tested the quiescence score in datasets of senescent cells (Hernandez-Segura et al.) and found that senescent cells also display high "quiescence scores." In fact, in two of the three datasets they show in Supplementary Figure 2a, the relative score differences in the medians between senescent and proliferating cells are even higher than those between quiescent and proliferating cells (as shown in almost all datasets in Supplementary Figure 1c). As stated in the response letter, the authors realized "our score captures a state of cell cycle arrest that is likely shared with other programmes such as senescence, stemness and dormancy."

That is, the "quiescent score" developed in this work is not sufficient to define quiescence but rather reflects cellular dormancy. This result needs to be clearly explained. The current version says, " the identification of quiescent cells within tumours presents an ongoing challenge …… quiescent tumour cells can be mistaken for other therapy resistant cell types such as senescent or cancer stem cells." "Biomarkers of quiescence that are sufficiently specific and robust to be clinically useful are clearly needed." However, given the current "quiescent score" identifies both quiescent and senescent cells but cannot distinguish them, it does not provide a sufficiently specific and robust matrix to identify quiescence cells to be clinically useful. That said, I think the score is still useful in identifying dormant cells — just not quiescent cells specifically. The authors need to thoroughly revise the paper, including the title, abstract, and main text, to clarify this critical point.

**Reviewer 2**

The authors have addressed most of my concerns and the manuscript has improved upon revision. There are several minor comments in regard to the response to Question #3 of the major comments.

1. In the CONCLUSIONS part, the author stated that "We propose a quiescence signature which can be robustly measured in bulk tissue or single cells, and could inform therapeutic strategies or risk of relapse". However, the new data provided in Supplementary Figures 7b-e showed that no clear relation was observed between quiescence and risk of relapse, which didn't support this statement. Thus, this needs to be deleted or rephrased or provide more data to support the statement.

2. The clinical significance of quiescence score needs further to be addressed in patients. Although the author showed that quiescence state was associated with drug resistance in several cell lines, no clear relation was observed between quiescence and the risk of relapse in patients. Could the authors provide additional data on the association between quiescence and treatment responses in patients? Specifically, are patients with higher quiescent score more likely to have poor response to some treatments?

3. In line 349-351, the author stated that "Indeed, such slow cycling, indolent tumors would have higher chances of being eradicated earlier in the disease, which is consistent with reported worse prognosis of patients with higher tumor cell proliferation rates and less stable, more mutated genomes(94)". There was paraphrase/misquotation of the conclusion in reference 94. Reference 94 reported that worse prognosis of patients with higher tumor cell proliferation. However, they didn't show the association between patient survival and genome instability.

**Authors Response**

**Point-by-point responses to the reviewers’ comments:**

Reviewer #1

The authors have addressed most of my previous technical questions. However, one critical issue remains. In the revision, the authors tested the quiescence score in datasets of senescent cells (Hernandez-Segura et al.) and found that senescent cells also display high "quiescence scores." In fact, in two of the three datasets they show in Supplementary Figure 2a, the relative score differences in the medians between senescent and proliferating cells are even higher than those between quiescent and proliferating cells (as shown in almost all datasets in Supplementary Figure 1c). As stated in the response letter, the authors realized "our score captures a state of cell cycle arrest that is likely shared with other programmes such as senescence, stemness and dormancy."

That is, the "quiescent score" developed in this work is not sufficient to define quiescence but rather reflects cellular dormancy. This result needs to be clearly explained. The current version says, " the identification of quiescent cells within tumours presents an ongoing challenge …… quiescent tumour cells can be mistaken for other therapy resistant cell types such as senescent or cancer stem cells." "Biomarkers of quiescence that are sufficiently specific and robust to be clinically useful are clearly needed." However, given the current "quiescent score" identifies both quiescent and senescent cells but cannot distinguish them, it does not provide a sufficiently specific and robust matrix to identify quiescence cells to be clinically useful. That said, I think the score is still useful in identifying dormant cells — just not quiescent cells specifically. The authors need to thoroughly revise the paper, including the title, abstract, and main text, to clarify this critical point.

*Reply: The Reviewer’s concern is highly justified, and we agree this needs to be properly clarified. We acknowledge that our signature does not uniquely distinguish quiescence from senescence in a scenario where either a deep quiescence or a senescence phenotype are both plausible, unless additional senescence-specific markers such as β-galactosidase are utilised. The similarities between these two phenotypes have been indeed reported in previous studies (Fujimaki and Yao, Physiol Genomics 2020; Fujimaki et al, PNAS 2019), which we have now more specifically commented on in the revised text (lines 163-166): “Indeed, we found that our signature could to a certain extent also separate senescent cells from proliferating ones in single cell data from Hernandez-Segura et al(55), which is unsurprising since these cells are also in the G0 phase (Supplementary Figure 2a). Indeed, the authors of this resource highlight that some of the pathways uncovered in these senescent cells may be shared with quiescence, which is also backed up by a study from Fujimaki and Yao(56) suggesting similarities between deep quiescence and senescence.” We have now expanded the discussion to touch upon this aspect, as highlighted in the last paragraph of this answer.*

*With respect to the terminology used for our signature, we have chosen to employ the term “quiescence” in the first place because our signature was derived and validated in experiments that were specifically tailored to induce and/or measure quiescence, and not dormancy or senescence. Therefore, we believe calling it a “quiescence signature” more accurately reflects its original derivation as well as its ability to capture reversible G0 arrest periods of varying duration. Even senescent cells are proposed to sometimes revert to a post-senescent, proliferative state by some studies (Deursen, Nature 2014, Zhang et al, Sci Rep 2018), and in this respect it could still be possible that our quiescence signature captured those cells which still maintained this capacity in the single cell datasets where it scored highly. Similarly, the definition of dormancy is contentious in itself, often being used interchangeably with quiescence, mostly in bacteria or plants (Lies et al, Yeast 2021, Rittershaus et al, Cell Host Microbe 2013), but not so often in cancer. The majority view in the cancer field refers to dormancy as metastatically disseminated cells residing in a non-proliferative state (Sosa et al, at Rev Cancer, 2014 [PMID: 25118602], Aguirre-Ghiso, Cancer Res 2021 [PMID: 34429327]). These dormant cells have been described in the literature to be in various stages of either quiescence, senescence, or slow-cycling (Davis et al, Adv Exp Med Biol, 2019 [PMID: 31576550]). Importantly, dormancy appears highly determined by microenvironmental factors (Ghajar et al , Nat Cell Biol 2013 [PMID: 23728425], Gao et al, BMC Cancer 2019 [PMID: 31357956]), rendering a cell cycle arrest phenotype as insufficient to define it.*

*Our manuscript refers to quiescent/non-cycling cells in the primary tumour which are not, by the clinical definition, in a state of dormancy. We had already made this distinction in the discussion previously (lines 559-562): “Importantly, the state of cancer quiescence that we have studied here is distinct from that of disseminated tumour cells causing clinical dormancy and cancer relapse, often after many years from the treatment of the primary tumour(6, 122)”. It is true that this cell cycle arrest state in primary tumours may also incorporate senescent cells, which should be taken into consideration. However, senescent cells are more likely to be cleared by the immune system (Prata et al, Semin Immunol 2018 [PMID: 31088710], Kale, Immunity and Ageing 2020 [PMID: 32518575]), so we assume the majority of remaining non-cycling cells in the pre-treated primary tumours which we are studying would be in a quiescent state. We also demonstrate in the manuscript in Supplementary Figures 2c-d that our signature does not reflect senescence in the pan-cancer TCGA datasets which we analyse. Therefore, when it comes to bulk pre-treatment data, the evidence points towards a quiescence phenotype, which would also be more likely biologically expected in primary tumours as a mechanism of adaptation during tumour growth and as response to stress. When it comes to the single cell data analysed, we have now further checked whether our single cell datasets show evidence of β-galactosidase expression, and this is not the case (SASP signals are less relevant in cell lines lacking the microenvironment, hence we did not check for them). We have now added this information in the text:*

*Lines 378 – 380: “Within the p53 proficient cell line RKO, the fraction of quiescent cells increased from 41% to 93% after treatment with a low dose (10 μM) of 5-FU and persisted at higher doses (Figure 6e-f). We confirmed these cells were indeed quiescent and not senescent, as β-galactosidase expression was depleted in these compared to their proliferative counterparts (0.4-fold depletion, Fisher’s exact test p<<0.001).”*

*Lines 394 – 396: “EGFR inhibition led to a massive decrease in cell numbers immediately after treatment, mostly due to proliferating cells dying off (Supplementary Figures 8c-d), while the proportion of quiescent cells increased to 96% at day 1, indicating an immediate selective advantage for such cells (Figure 6i-j). These cells are not senescent (depletion of β-galactosidase expressing cells compared to the proliferative group, Fisher’s exact test p<<0.001) and appear to gradually start proliferating again in the following days during continuous treatment.”*

*Thus, we are confident all the findings reported in the manuscript pertain to quiescence rather than senescence phenotypes. We have, however, now added a sentence in the discussion to emphasise the importance of testing for β-galactosidase expression to exclude the possibility that a senescent phenotype might be surveyed instead when using our signature (lines 486-488): “In the meantime, future studies utilising our quiescence signature should also test for β-galactosidase activity to exclude the possibility of a senescent state being captured instead.”*

*We are cautious about not making any claims around dormancy given the rather varied and sometimes ambiguous definitions in the field, which are mostly due to our limited understanding of this highly complex phenotype. The main focus of our manuscript is on the role of quiescence within primary tumours and as enablers of therapeutic resistance, and characterising other related states is outside the scope of this study. However, we acknowledge the lack of clarity in the definitions and the potential overlap between these states has not been sufficiently discussed in the manuscript and have now expanded the Discussion section to further comment on these aspects (lines 472-488):*

*“Indeed, gene activity linked to cell cycle arrest is not exclusive to quiescence, but can be shared with senescence or dormancy in certain scenarios, as also demonstrated in some of our analyses of senescent cells. This makes it difficult to clearly distinguish states like dormancy, senescence, quiescence (particulary deep quiescence), as even their definitions can be contentious at times both in the context of human cancers (6, 56, 106-108) as well as in physiological conditions in other organisms (109, 110). However, since our signature was derived and validated in experiments that were tailored specifically to induce and/or measure quiescence, we believe the signature proposed in this study best reflects a quiescent-like, reversible G0 cell cycle arrest state. We have also ensured throughout the study that the states we were examining in bulk and single cell samples did not coincide with senescence, and thus any findings reported here can be considered as pertaining to the quiescence phenotype. While senescent and dormant cells could be distinguished from their quiescent counterparts simply based on additional senescence and dormancy markers, further research is nevertheless required in the future to delineate signatures that are both necessary and sufficient to unambiguously discriminate all three states. In the meantime, future studies utilising our quiescence signature should also test for β-galactosidase activity to exclude the possibility of a senescent state being captured instead.”*

Reviewer #2:

The authors have addressed most of my concerns and the manuscript has improved upon revision. There are several minor comments in regard to the response to Question #3 of the major comments.

1. In the CONCLUSIONS part, the author stated that "We propose a quiescence signature which can be robustly measured in bulk tissue or single cells, and could inform therapeutic strategies or risk of relapse". However, the new data provided in Supplementary Figures 7b-e showed that no clear relation was observed between quiescence and risk of relapse, which didn't support this statement. Thus, this needs to be deleted or rephrased or provide more data to support the statement.

The link between our quiescence signature and risk of relapse was a hypothetical statement assuming longer-term research into the topic. The Reviewer makes a very good point that there is insufficient evidence for any direct link to relapse from the current study, and we apologise for the oversight in amending this statement in the first place. We have now removed this part and the new sentence reads as follows (line 572): “We propose a quiescence signature which can be robustly measured in bulk tissue or single cells, and could potentially inform therapeutic strategies in the longer term.”

2. The clinical significance of quiescence score needs further to be addressed in patients. Although the author showed that quiescence state was associated with drug resistance in several cell lines, no clear relation was observed between quiescence and the risk of relapse in patients. Could the authors provide additional data on the association between quiescence and treatment responses in patients? Specifically, are patients with higher quiescent score more likely to have poor response to some treatments?

*Reply: We thank the Reviewer for emphasising the need to further discuss/evidence the link between quiescence and therapy response in actual patient cohorts. While datasets with RNA-sequenced patient samples and well annotated response data are quite scarce, we have further analysed the TCGA data to link quiescence with response to therapy, as well as a few independent GEO datasets, including several from the SELECT study (Lee et al, Cell 2021), the results of which are now included in a new Supplementary Figure 10. While we see some evidence for pre-existing quiescence in the primary tumours being linked with residual disease after neoadjuvant chemotherapy in a breast cancer study, this does seem to be generalisable. In fact, in most TCGA and SELECT cohorts we see no link between quiescence and treatment response, and occasionally quiescence may be linked with better outcomes – but this is highly tissue and treatment specific. This is described as follows:*

*Results (lines 416-429): “While links between quiescence and therapeutic resistance are prevalently observed in cell lines, one would question whether this translates to similar pathology in cancer patients. While we observed significantly higher quiescence levels in pre-treated tumours of non-responders to neoadjuvant chemotherapy in a breast cancer study by Hatzis et al(102) (Supplementary Figure 10a), surveying various targeted therapy datasets from the SELECT study(103) and the TCGA data for links with response to various therapies (single agent and combinations) showed little to no evidence for a bulk signature of quiescence being useful for predicting resistance in these studies (Supplementary Figures 10b-c). Although the studies available for inspection are rather sparse, evidence from all the analyses presented here suggests there is no universal a priori role that quiescence has within the pre-treated primary tumour in determining response to treatment, with more favourable overall outcomes observed occasionally due to slower progressing malignancy, but also resistance observed in the case of chemotherapy in breast cancer. Instead, the role of quiescence in enabling therapeutic resistance as a short-lived acquired phenotype as demonstrated in single cell datasets appears more consistent.”*

*Methods (lines 305-308):”Datasets have been obtained from the GEO database through the following GEO Series accession numbers […] for the patient sample datasets: GSE191127, GSE109211, GSE50509, GSE65185, GSE66399, GSE68871, GSE99898 (Supplementary Table 3). Unified treatment response data for TCGA was obtained from Moiso, medRxiv 2021(146).”*

*Therefore, we suggest it is unlikely that pre-existing quiescence can be generalised as a marker for therapeutic response, but instead seems to be highly context specific and requires further research. The analyses we have performed to date seem to suggest that quiescence is an enabler of therapeutic resistance in multiple scenarios, but it is a short-lived phenotype in this context and most likely acquired rather than pre-existing (although we currently lack the experimental setup to test this, which is why we have not speculated too much in the discussion). This is further backed up by the observation that high levels of quiescence across the entire tumour in the primary seem to be linked with better outcomes. Thus, the highest utility of our signature we believe lies in its capacity to specifically mark quiescent cells and thus enable further detailed studies of how therapeutic resistance emerges, rather than acting as a predictive marker. We have expanded the Discussion to clarify these points (lines 539-550):*

*“Given the variable links to treatment response and lack of clear evidence for relapse when surveying quiescence in primary tumours before treatment, it is likely our quiescence signature in its current form cannot be used to predict resistance to chemotherapy or targeted therapy, and we would not recommend it for this purpose unless further validated in a specific cancer setting. We have also not inspected the role of quiescence in the context of immunotherapy, which remains an area of future study. However, we believe our signature has high value for the study of emerging resistance in an in vitro/in vivo setting, as a short-lived enabler of drug tolerance. The optimised signature we propose for single cell data makes it tractable to a variety of future studies in this area.”*

3. In line 349-351, the author stated that "Indeed, such slow cycling, indolent tumors would have higher chances of being eradicated earlier in the disease, which is consistent with reported worse prognosis of patients with higher tumor cell proliferation rates and less stable, more mutated genomes(94)". There was paraphrase/misquotation of the conclusion in reference 94. Reference 94 reported that worse prognosis of patients with higher tumor cell proliferation. However, they didn't show the association between patient survival and genome instability.

*Reply: We thank the Reviewer for pointing out this mistake. The paper was indeed misquoted and we have now amended this. We have removed any claims regarding how genomic instability may be linked to progression, as the relation here is not an entirely straightforward one but may depend on the extent of genomic instability (Andor et al, Cancer Research 2018). Nevertheless, this does not detract in any way from our original finding, which was simply about the link between cell proliferation rate and prognosis. The amended sentence reads as follows (lines 346-349): “Indeed, such slow cycling, indolent tumors would have higher chances of being eradicated earlier in the disease, which is consistent with reported worse prognosis of patients with higher tumor cell proliferation rates (95).”*

**Third round of review**

**Reviewer 1**

I appreciate the detailed explanation by the authors to address my concerns raised in the previous round. However, I am sorry to say that I cannot agree with the authors this time. First, "because our signature was derived and validated in experiments that were specifically tailored to induce and/or measure quiescence" does not justify calling it a "quiescence signature." Indeed, quiescent cells are captured by this signature - the problem is those captured by this signature are not necessarily quiescent - they can be senescent instead, as seen in Supplementary Figure 2a.

Second, the authors said they "ensured throughout the study that the states we were examining in bulk and single cell samples did not coincide with senescence, and thus any findings reported here can be considered as pertaining to the quiescence phenotype." The evidence they presented was that β-galactosidase expression was depleted in these cells compared to their proliferative counterparts. However, it has been well known and cited that although it remains the most widely used senescent biomarker for its convenience, SA-β-gal is dispensable and not required for senescence induction and maintenance (https://doi.org/10.1111/j.1474-9726.2006.00199.x). Therefore, lacking SA-β-gal expression does not indicate the cells are quiescent and not senescent.

It is misleading to call the quiescence score developed in this work a "quiescence signature", when it cannot differentiate quiescent from senescent cells. The authors presented it as a challenge upfront that "quiescent tumour cells can be mistaken for other therapy resistant cell types such as senescent cells." Unfortunately, this "quiescence signature" presented here did not solve this problem and thus may not inform therapeutic strategies as we hoped.

**Reviewer 2**

The authors have addressed all of my concerns and the manuscript has improved upon revision.

**Authors Response**

**Point-by-point responses to the reviewers’ comments:**

Reviewer #1:

I appreciate the detailed explanation by the authors to address my concerns raised in the previous round. However, I am sorry to say that I cannot agree with the authors this time. First, "because our signature was derived and validated in experiments that were specifically tailored to induce and/or measure quiescence" does not justify calling it a "quiescence signature." Indeed, quiescent cells are captured by this signature - the problem is those captured by this signature are not necessarily quiescent - they can be senescent instead, as seen in Supplementary Figure 2a.

Second, the authors said they "ensured throughout the study that the states we were examining in bulk and single cell samples did not coincide with senescence, and thus any findings reported here can be considered as pertaining to the quiescence phenotype." The evidence they presented was that β-galactosidase expression was depleted in these cells compared to their proliferative counterparts. However, it has been well known and cited that although it remains the most widely used senescent biomarker for its convenience, SA-β-gal is dispensable and not required for senescence induction and maintenance (https://eur01.safelinks.protection.outlook.com/?url=https%3A%2F%2Fdoi.org%2F10.1111%2Fj.1474-9726.2006.00199.x&data=05%7C01%7Cm.secrier%40ucl.ac.uk%7Cda351e2648024e36bc9808daec01a2da%7C1faf88fea9984c5b93c9210a11d9a5c2%7C0%7C0%7C638081789018602680%7CUnknown%7CTWFpbGZsb3d8eyJWIjoiMC4wLjAwMDAiLCJQIjoiV2luMzIiLCJBTiI6Ik1haWwiLCJXVCI6Mn0%3D%7C3000%7C%7C%7C&sdata=NqPncKvr%2BXvmJ%2FdUT5tAxxrVhibq8X%2Faq8H1u4HxcMI%3D&reserved=0). Therefore, lacking SA-β-gal expression does not indicate the cells are quiescent and not senescent.

It is misleading to call the quiescence score developed in this work a "quiescence signature", when it cannot differentiate quiescent from senescent cells. The authors presented it as a challenge upfront that "quiescent tumour cells can be mistaken for other therapy resistant cell types such as senescent cells." Unfortunately, this "quiescence signature" presented here did not solve this problem and thus may not inform therapeutic strategies as we hoped.

*Reply: We thank the Reviewer for further clarifying their concerns regarding the ambiguity of cell cycle arrest state that is captured in some of the analyses in this study. We appreciate the opportunity to reflect on this aspect further, and agree that lack of strong evidence for a particular phenotype (especially when its associated markers are quite variable) does not exclude that phenotype altogether. The reviewer is right that these G0 arrest states cannot be unambiguously distinguished using our signature. We have therefore renamed the state that we are studying to “G0 arrest”, which we believe correctly encompasses different quiescence/senescence/dormancy scenarios while at the same time providing a more accurate and more useful interpretation of the findings. We have revised the title of the manuscript to “Genomic hallmarks and therapeutic implications of G0 cell cycle arrest in cancer” and the wording to “G0 arrest” throughout the abstract, main manuscript, figures and supplementary material (please see tracked changes in the main manuscript). We have also slightly rephrased some of the discussion points on identification of these states and association to therapeutic resistance to better reflect this broader G0 state (see tracked changes in lines 69-82, 686-693). These changes do not alter any of the key messages of the paper, but they help clarify that we are fundamentally looking at a G0 arrest state which is broadly relevant for understanding cancer progression and therapeutic resistance in a variety of scenarios.*

*This is a genuinely fairer reflection of the range of phenotypes our signature captures, as it can flexibly reflect longer or shorter cell cycle arrest states depending on the strength of the score, and thus could indeed also identify senescent and dormant cells. We are grateful to the Reviewer for enabling us to appreciate this and for the opportunity to make this change, which helps emphasise the relevance of our work beyond just quiescence. Indeed, we had suggested before that our signature and findings could be used as a baseline to study different cell cycle arrest states, from quiescence to senescence to dormancy, and the current terminology makes this much clearer. Thus, we believe this change will also increase the appeal and relevance of our work to the wider community studying different cell cycle arrest states in cancer.*

**Fourth round of review**

**Reviewer 1**

The authors have addressed all of my main questions. I congratulate the authors on this manuscript.
